# Does Pre-existing Diabetes Correlate with Long COVID-19 in Europe? Evidence from the Analysis of the Survey of Health, Ageing and Retirement in Europe's Corona Surveys

**DOI:** 10.1155/2024/7459628

**Published:** 2024-02-02

**Authors:** Sarah Cuschieri, Piotr Wilk

**Affiliations:** ^1^Faculty of Medicine and Surgery, University of Malta, Msida, Malta; ^2^Department of Epidemiology and Biostatistics, Western University, London, Canada; ^3^Department of Epidemiology, Maastricht University, Maastricht, Netherlands

## Abstract

**Background:**

A substantial proportion of those infected with COVID-19 are presenting with persistent symptoms, referred to as long COVID-19. Emerging evidence suggests that the presence of pre-existing chronic conditions, such as diabetes, may increase the risk of long COVID-19.

**Objectives:**

To investigate whether having pre-existing diabetes increases the risk of developing long COVID-19 in the population of middle-aged and older adults (≥50 years old) in Europe, while assessing if this relationship can be accounted for or is modified by the known long COVID-19 and diabetes risk factors (age, sex, hospitalization, pre-existing hypertension, and weight status).

**Methods:**

A population-based longitudinal prospective study involving a sample of respondents aged 50 years and older (*n* = 4,004) with probable or confirmed COVID-19 infection from 27 countries that participated in both waves 7 and 8 of the Survey of Health, Ageing and Retirement in Europe and its 2020 and 2021 Corona Surveys. Logistic regression modeling was performed.

**Results:**

Overall, 66.8% of the respondents affected by COVID-19 infection reported at least one long COVID-19 symptom; 55.2% were female, and the average age was 64.6 years; 13.2% had pre-existing diabetes. Respondents with pre-existing diabetes had significantly higher odds of developing long COVID-19, compared to those without diabetes (OR = 1.37; 95% CI = 1.12, 1.68). This relationship remained significant (OR = 2.00; 98% CI = 0.25, 1.14) after adjusting for sex (OR = 1.64 for females; 95% CI = 1.43, 1.88), hospitalization for COVID-19 illness (OR = 3.19; 95% CI = 2.41, 4.23), pre-existing hypertension (OR = 1.17; 95% CI = 1.01, 1.36), and overweight (OR = 1.31; 95% CI = 1.11, 1.56) and obese (OR = 1.77; 95% CI = 1.44, 2.19) weight status. The effect of pre-existing diabetes on the risk of long COVID-19 is moderated by age; it was highest at the age of 50 (OR = 2.00; 95% CI = 1.28, 3.14), and then, it declined with age.

**Conclusions:**

There is a relationship between pre-existing diabetes and long COVID-19, even after controlling for literature-based confounding factors, with age having a moderating effect on this relationship.

## 1. Background

The coronavirus disease 2019 (COVID-19) outbreak, a global pandemic since early 2020, resulted in a substantial increase in morbidity and mortality among different populations. Following the acute stage of a COVID-19 infection, over 60% of Europeans continue to experience symptoms in a phenomenon referred to as long COVID-19 [1]. Long COVID-19 has been defined by the World Health Organization (WHO) as a condition that occurs in those with probable or confirmed COVID-19 infection where symptoms last for at least three months from the onset of the infection [2]. This is a multisystem disease with a long-lasting inflammatory mechanism and potential for organ damage, although research is still ongoing [3]. There is strong evidence that women, older adults, individuals with overweight or obese weight status, and those requiring hospitalization during the acute phase of their COVID-19 infection are at a higher risk of developing long COVID-19 [4–6]. Emerging evidence suggests that the presence of pre-existing chronic conditions such as diabetes may also increase the risk of developing long COVID-19 [5, 7–9].

Diabetes is a global epidemic that has been dominating the scene for much longer than the onset of COVID-19 [10]. Contributing risk factors for the development of diabetes have been well established and include an overweight or obese weight status; older age; African, American, and Asian ethnocultural background; and history of hypertension or cardiovascular disease [11]. Individuals with diabetes have a higher susceptibility to infection, especially in the presence of hyperglycaemia, with enhanced infection severity and risk of mortality [12, 13]. These individuals exhibit chronic low-grade inflammation as a result of impaired insulin signaling [14]. On acquiring COVID-19, individuals with diabetes have been noted to sustain a more severe infection [15]. An acute COVID-19 infection leads to metabolic deterioration, while an individual with diabetes already has an underlying chronic inflammatory state with changes in the immune system. Therefore, on COVID-19 infection, individuals with diabetes are anticipated to have a higher risk of developing long COVID-19 [8]. However, emerging evidence on the role of pre-existing diabetes on the susceptibility to experience long COVID-19 is contradictory, and further research has been recommended [5, 16, 17].

Thus, the objective of this study was to investigate whether having pre-existing diabetes increases the risk of developing long COVID-19 in the population of middle-aged and older adults (≥50 years old) in Europe. We also assessed if this relationship can be accounted for or is modified by the known long COVID-19 risk factors (i.e., age, sex, and COVID-19-related hospitalization) as well as common risk factors for diabetes and long COVID-19 (i.e., weight status and hypertension). Such evidence will enable physicians and public health officials to deliver targeted preventive action strategies while gaining a better understanding of long COVID-19 and the role of pre-existing diabetes.

## 2. Methods and Materials

### 2.1. Sample

This study employed data from waves 7 (2017) and 8 (2019-2020) of the main SHARE survey [18] as well as data from the SHARE's 2020 and 2021 Corona Surveys [19, 20] that were collected from a large cohort of middle-aged and older adults in response to the COVID-19 pandemic, in 27 countries across Europe (*n* = 47,964). These longitudinal surveys provide an opportunity to examine the impact of prepandemic characteristics, including pre-existing chronic conditions on the risk of COVID-19 infection and, subsequently, long COVID-19. The study sample consisted of 4,004 individuals aged 50 years and older who, up to 12 months before the Corona Survey 2 (summer 2021), indicated that they either had a positive test for the SARS-CoV-2 virus (*n* = 3,103) or experienced symptoms that they attributed to a COVID-19 infection but were not tested (*n* = 901).

### 2.2. Measurements

Survey participants were asked if they experienced at least one of the following nine long-term or lingering effects that they attributed to their COVID-19 illness: fatigue; cough, congestion, shortness of breath; loss of taste or smell; headache; body aches and joint pain; chest or abdominal pain; diarrhea and nausea; confusion; or any other symptoms. Respondents were considered to have long COVID-19 if they reported to have one or more of these symptoms. Information on self-reported diabetes and hypertension was obtained from the Corona Survey 1 or from waves 7 and 8 of the main SHARE survey, depending on when the respondents reported these conditions. The survey questionnaire did not differentiate between the types of diabetes respondents were suffering from. Based on their responses to these surveys, individuals were categorized as “having pre-existing diabetes” or “having pre-existing hypertension” (vs. “not having diabetes” or “not having hypertension,” respectively). For 69 individuals (1.7%) with missing data on their diabetes status, we used their responses from the Corona Survey 2. Based on their responses to waves 7 or 8 of the main SHARE survey, we categorized respondents as being “overweight” or “obese” (vs. “underweight/normal weight”). Hospitalization related to COVID-19 illness was measured at the time of the Corona Survey 2. Age was measured as a continuous variable (centered at 50 years), and sex was specified as a binary indicator (female vs. male).

### 2.3. Data Analysis

To assess the association between pre-existing diabetes and long COVID-19 in a sample of individuals affected by COVID-19 infection, we ran two logistic regression models: an unadjusted model to estimate the overall relationship between these two health conditions (model 1) and an adjusted model, where we controlled for known risk factors for diabetes and long COVID-19 (i.e., age, sex, hospitalization, hypertension, and weight status) (model 2). In the second model, we also explored modifying effects (i.e., interactions) of these risk factors on the effect of diabetes on long COVID-19. A *p* value of 0.05 or less was used in all tests. There were 121 (3.0%) individuals with missing data points, including 13 (0.3%) for long COVID-19 and 98 (2.4%) for weight status. We performed 50 imputations to address this problem. A supplementary Table S1 provides descriptive statistics for individuals with (*n* = 121) and without (*n* = 3,883) any missing points; it suggests that those with any missing data points were, on average, younger and less likely to have hypertension. We used calibrated cross-sectional sampling weights from the Corona Survey 2. All analyses were conducted in SAS 9.4 [21].

## 3. Results

Descriptive statistics for the sample of 4,004 respondents aged 50 years and older who were previously infected with COVID-19 are presented in Table 1, and they include unweighted frequencies and weighted percentages. Overall, 66.8% of the respondents reported having at least one symptom related to long COVID-19; 55.2% were female, and the average age was 64.6 years. In total, 13.2% of the respondents indicated that they were diagnosed with diabetes before their COVID-19 illness. This proportion was larger among those who subsequently reported long COVID-19 (14.4%) than among those who did not develop long COVID-19-related symptoms (10.9%). In addition, 11.3% of the respondents indicated that they were hospitalized due to their COVID-19 illness and 40.6% were diagnosed with hypertension before their COVID-19 infection. Based on the information collected before the COVID-19 pandemic, 43.1% of respondents were overweight and 23.8% were obese, compared to 33.1% who reported having normal weight status.

**Figure 1:**
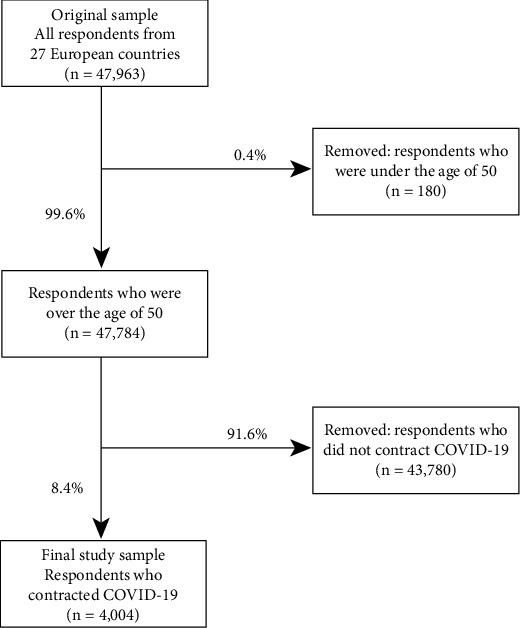
Flowchart illustrating the inclusion and exclusion criteria for the selected study sample.

Parameter estimates, including odds ratios (OR) and their 95% confidence intervals (CI), for the two logistic regression models are reported in Table 2. The results from the unadjusted model suggest that individuals with pre-existing diabetes had significantly higher odds of having long COVID-19, compared to those who were not diagnosed with diabetes before their COVID-19 infection (OR = 1.37; 95% CI = 1.12, 1.68); that is, they had a 37% higher risk of reporting at least one symptom related to long COVID-19. In the second model, when we adjusted for the effects of sex, age, hospitalization, hypertension, and weight status, the association between pre-existing diabetes and long COVID-19 remained statistically significant, although it was modified by age. Specifically, among individuals with pre-existing diabetes, the risk of long COVID-19 was highest at the age of 50 (OR = 2.00; 95% CI = 1.28, 3.14) and declined with age at a rate of 23% per 10 years (OR = 0.77; 95% CI = 0.61, 0.96).  Figure 1 illustrates the nature of this relationship in terms of predicted probabilities for a modal group of respondents (i.e., nonhospitalized females of normal weight status without hypertension). It shows that among those with pre-existing diabetes, the proportion of individuals estimated to have long COVID-19 decreased from 78% at the age of 50 to 43% at the age of 90, compared to a less pronounced decrease from 64% to 59% among individuals without diabetes. The results for other interaction effects with pre-existing diabetes suggest that sex (chi − square = 0.35; *p* = 0.55), hospitalization (chi − square = 0.01; *p* = 0.93), hypertension (chi − square = 0.03; *p* = 0.87), and weight status (chi − square = 5.52; *p* = 0.06) did not have a statistically significant modifying effect on the relationship between pre-existing diabetes and long COVID-19.

In terms of the role of the other risk factors for long COVID-19 that we assessed in this study, the results from the second model suggest that, controlling for other risk factors, females (OR = 1.64; 95% CI = 1.43, 1.88), respondents who were hospitalized for COVID-19 illness (OR = 3.19; 95% CI = 2.41, 4.23), and those with pre-existing hypertension (OR = 1.17; 95% CI = 1.01, 1.36) were more likely to report at least one long COVID-19-related symptom. Finally, individuals with overweight (OR = 1.31; 95% CI = 1.11, 1.56) and obese (OR = 1.77; 95% CI = 1.44, 2.19) weight status were at higher risk of developing long COVID-19, compared to individuals with normal weight status.

## 4. Discussion

There is lack of consistent evidence on whether the presence of pre-existing chronic conditions increases the risk of developing long COVID-19. This study was set to explore the relationship between pre-existing diabetes and long COVID-19 among middle-aged and older adult (≥50 years old) residents of Europe.

In general, individuals with diabetes are more susceptible to infections, including COVID-19 [12, 22]. Thus, when affected by COVID-19, they could have a worse infection through three potential pathways: (i) hyperglycaemia resulting in oxidative stress and inflammation, (ii) hyperglycaemia leading to the inhibition of lymphocyte proliferation, and (iii) binding of SARS-CoV-2 to pancreatic acinar cells leading to tissue damage [15]. Therefore, we anticipated that pre-existing diabetes is also linked to an increased risk of having long COVID-19 symptoms. This hypothesis was confirmed in our unadjusted model where without considering other risk factors related to diabetes and long COVID-19, individuals with pre-existing diabetes were found to be 37% more likely to have long COVID-19 than those without diabetes. This finding supports a recent literature review that noted that individuals with diabetes may be more susceptible to long COVID-19 [23].

We also found that the relationship between pre-existing diabetes and long COVID-19 remained statistically significant, after we controlled for known diabetes and long COVID-19 risk factors, gender, age, hospitalization, hypertension, and weight status [5, 24–27]. However, when we explored the possibility that these risk factors may affect individuals with pre-existing diabetes differently than those without this chronic condition, we found that, although individuals with diabetes are, overall, more likely to have long COVID-19, this risk varies by age. Specifically, middle-aged adults, up to age 70, with diabetes, are more likely to have long COVID-19 than their peers not affected by this chronic condition. However, from age 70 onward, individuals with pre-existing diabetes are less likely to report any symptoms related to long COVID-19 than those without diabetes.

The following are the key implications of these findings:

1. Overall, pre-existing diabetes is a risk factor for long COVID-19, without and with adjustment for other risk factors
2. However, the risk of long COVID-19 changes significantly (and substantially) across the age groups, with older adults (after age 70) with diabetes being less likely to have long COVID-19 than those without diabetes. Among middle-aged adults, those with diabetes are more likely to have long COVID-19 than those without diabetes. Some past studies involving population-based studies suggest that the relationship between age and long COVID-19 is nonlinear with older adults being at lower risk of long COVID-19 [28]. However, none of these studies explored if this decline in the risk is homogenous across all individuals. The results of our study suggest that the decline in the risk of long COVID-19 is much more pronounced among those with pre-existing diabetes. This may be arising from the fact that older adults with pre-existing diabetes might have been suffering symptoms, such as fatigue, that may be similar to common long COVID-19 symptoms for a substantial period of time. Hence, these individuals are more likely to be acquainted with these persistent symptoms and less likely to report them as long COVID-19 related. That is, they may have attributed long COVID-19 symptoms to their pre-existing diabetes

In terms of the previously reported risk factors for long COVID-19, female sex has been linked with a higher risk of developing long COVID-19 [24, 26] due to a stronger immune response (both innate and adaptive) when compared to males. While this protects females from the initial infection, it renders them more susceptible to prolonged related autoimmune diseases [29–31]. Another aspect to consider is the potential overlapping symptoms of perimenopause and menopause with those of long COVID-19, especially considering that older age is another risk factor for long COVID-19 [24, 32]. It needs to be noted that although males tend to have a higher susceptibility to the development of diabetes, psychosocial stress and older age predispose females to the development of diabetes [33–35].

It is known that females tend to have a higher body fat composition than males, predisposing them to diabetes [36], which might explain the positive relationships established in our study between long COVID-19 symptoms with female sex and overweight and obese weight status. This coincides with the evidence where females and high BMI were associated with long COVID-19 [24, 37]. The underlying pathophysiology for this has been attributed to the association of obesity with systemic inflammation originating from multisystemic states (metabolic, hormonal, and proinflammatory) [38]. Evidence has also linked pre-existing hypertension and development of long COVID-19 symptoms [39]. It needs to be noted that both an overweight and obese weight status and hypertension are also risk factors for the development of diabetes [11]. Therefore, it appears that long COVID-19 and pre-existing diabetes share similar risk factors, as noted in this study.

### 4.1. Study Strengths and Limitations

The data used in this study were obtained from the Survey of Health, Ageing and Retirement in Europe (SHARE), a population-based longitudinal research infrastructure, which plays as a strength in this study since the results represent the European ecosystem. Additionally, the study sample was made up of both individuals requiring COVID-19-related hospitalization (over 11%) and those that did not, which might explain the general (unadjusted) positive association between pre-existing diabetes and long COVID-19, as opposed to literature [16]. Our study has several limitations. The definition of COVID-19 infection was based on a prior reporting of having been tested for SARS-CoV-2 virus or else having experienced symptoms attributed to COVID-19. This definition might be subject to selection bias as those asymptomatic or experiencing a mild infection might not be represented. The definition of long COVID-19 and the respective estimations were assumed to reflect the prevalence of symptoms “up to 12 months” since the infection onset. Although in Corona Survey 2, the participants were asked to report long-term symptoms following the COVID-19 illness that occurred after their participation in Corona Survey 1, participants were not requested to provide any information on the infection date nor symptom duration or severity. Therefore, although our definition and assumptions on the follow-up are reasonable, the reported symptoms may be attributed to those of the acute phase of their infection or initiated before the WHO's recommendation of the three-month interval. Consequently, our results might be overestimating the prevalence of long COVID-19. Additionally, self-reported symptoms that were attributed to long COVID-19 might have overlapped with symptoms arising from other underlying health conditions resulting in an overestimation of long COVID-19 prevalence.

The definition used for pre-existing diabetes seems reasonable since the data were collected before the COVID-19 illness. However, we cannot exclude that some participants developed diabetes following a COVID-19 infection that took place after Corona Survey 1; hence, an overestimation of pre-existing diabetes prevalence may have been present. The lack of data on the subtypes of diabetes precluded us from investigating their impact on long COVID-19. This is a limitation, and it is recommended that further research is conducted to assess differences between individuals diagnosed with different types of diabetes.

The risk factors and confounders considered in our study were limited to those reported in the existing literature and available in SHARE. However, other factors, such as duration of diabetes and lifestyle factors, might be playing a role in this relationship. It is recommended that prospective cohort studies targeting individuals with different types of diabetes are carried out to close the knowledge gap. These studies, for instance, could collect life history calendar data on the onset and development of diabetes and on the relevant behavioral risk factors. These could serve as the basis for monitoring the effects of future outbreaks of COVID-19-related infections. Finally, as in other population-based surveys, the data are self-reported and subject to self-report bias, recall bias, and measurement error.

## 5. Conclusion

Our results suggest that there is a relationship between pre-existing diabetes and long COVID-19, even after controlling for literature-based confounding factors. We also found that age modifies this relationship. It is therefore recommended that prospective cohort studies are conducted to determine whether the long-term effects of COVID-19 are further complicated by pre-existing diseases, such as diabetes, or are a direct continuation of COVID-19. This will enable better understanding of whether pre-existing chronic conditions are contributing to the development of long COVID-19 which is known to exacerbate the health system burden and to negatively affect quality of life of affected individuals.

## Figures and Tables

**Table 1:** Descriptive statistics for study variables by long COVID-19 status.

Variable	Category	Frequency (*n*) (unweighted)	Percent (%) (weighted^∗^)	With long COVID-19 (%) (weighted^∗^)	Without long COVID-19 (%) (weighted^∗^)
Long COVID-19	No	1,118	33.2		
	Yes	2,873	66.8		
	Missing	13			

Diabetes	No	3,425	86.8	85.6	89.1
	Yes	579	13.2	14.4	10.9

Sex	Male	1,641	44.9	41.6	51.5
	Female	2,363	55.2	58.5	48.5

Age	<59	651	40.0	41.0	38.2
	60-69	1,779	34.2	32.8	36.7
	70-79	1,134	16.9	17.0	16.6
	80+	440	8.9	9.2	8.5
	Mean [SD]	68.0 [8.6]	64.6 [9.2]	64.5 [8.9]	64.7 [9.9]

Hospitalized	No	3,484	88.7	85.5	95.1
	Yes	508	11.3	14.5	4.9
	Missing	12			

Hypertension	No	2,140	59.4	57.1	64.1
	Yes	1,848	40.6	42.9	35.9
	Missing	16			

Weight status	Normal/underweight	1,144	33.1	30.2	38.3
	Overweight	1,594	43.1	43.0	43.5
	Obese	1,168	23.8	26.8	18.2
	Missing	98			

Source: SHARE, Corona Survey 1 and 2, and SHARE waves 7 and 8 (*n* = 4,004). ^∗^Calibrated cross-sectional sampling weights from the Corona Survey 2 were used. SD: standard deviation.

**Table 2:** Results from the unadjusted and adjusted logistic regression models.

Model	Parameter	Estimate	SE	*p* value	OR	CI—low	CI—high
Unadjusted	Intercept	0.66	0.04	<0.0001	1.93	1.80	2.07
	Diabetes	0.32	0.10	0.002	1.37	1.12	1.68

Adjusted	Intercept	0.09	0.10	0.359	1.09	0.90	1.32
	Diabetes	0.70	0.23	0.002	2.00	1.28	3.14
	Sex (female)	0.49	0.07	<0.0001	1.64	1.43	1.88
	Age (10 years)	-0.06	0.04	0.178	0.95	0.87	1.03
	Hospitalized	1.16	0.14	<0.0001	3.19	2.41	4.23
	Hypertension	0.16	0.08	0.041	1.17	1.01	1.36
	Overweight	0.27	0.09	0.002	1.31	1.11	1.56
	Obese	0.57	0.11	<0.0001	1.77	1.44	2.19

Interaction	Diabetes × age	-0.33	0.11	0.003	0.72	0.58	0.89

Source: SHARE, Corona Survey 1 and 2, and SHARE waves 7 and 8 (*n* = 4,004) with calibrated cross-sectional sampling weights from the Corona Survey 2. OR: odds ratio; CI: confidence interval; SE: standard error.

## Data Availability

Data is available from the SHARE project's website: https://share-eric.eu/.

## Abbreviations

COVID-19: Coronavirus disease 2019
OR: Odds ratio.

## Supplementary Materials

Supplementary Materials The effect of missing data has been assessed across different variables and has been presented in Supplement Table 1.
