# Supplementary material for: Does Pre-existing Diabetes Correlate with Long COVID-19 in Europe? Evidence from the Analysis of the Survey of Health, Ageing and Retirement in Europe's Corona Surveys
Source: J Diabetes Res. 2024 Feb 2;2024:7459628. doi: 10.1155/2024/7459628 (PMC10857882; doi:10.1155/2024/7459628)
Supplement: Supplementary Materials — The effect of missing data has been assessed across different variables and has been presented in Supplement Table 1. [file 7459628.f1.docx]

| **Table S1** | **Assessment of the effects of missing data** | |  |  |  |  |  |  |
| --- | --- | --- | --- | --- | --- | --- | --- | --- |
|  |  |  |  |  |  |  |  |  |
|  |  |  |  |  |  |  |  |  |
| **Variable** | **Category** | **No missing data points (n=3,883)** | | **Any missing data points (n=121)** | | **Chi-Square** | **p-value** |  |
|  |  | (n) | (%) | (n) | (%) |  |  |  |
|  |  |  |  |  |  |  |  |  |
|  |  |  |  |  |  |  |  |  |
| Long Covid | No | 1,080 | 96.6 | 38 | 3.4 | 2.83 | 0.0924 |  |
|  | Yes | 2,803 | 97.6 | 70 | 2.4 |  |  |  |
|  |  |  |  |  |  |  |  |  |
| Diabetes | No | 3,318 | 96.9 | 107 | 3.1 | 0.84 | 0.3586 |  |
|  | Yes | 565 | 97.6 | 14 | 2.4 |  |  |  |
|  |  |  |  |  |  |  |  |  |
| Sex | Male | 1,592 | 97.0 | 49 | 3.0 | 0.01 | 0.9117 |  |
|  | Female | 2,291 | 97.0 | 72 | 3.1 |  |  |  |
|  |  |  |  |  |  |  |  |  |
| Age | < 59 | 621 | 95.4 | 30 | 4.6 | 10.60 | 0.0141 |  |
|  | 60 - 69 | 1,721 | 96.7 | 58 | 3.3 |  |  |  |
|  | 70 - 79 | 1,111 | 98.0 | 23 | 2.0 |  |  |  |
|  | 80 + | 430 | 97.7 | 10 | 2.3 |  |  |  |
|  |  |  |  |  |  |  |  |  |
| Hospitalized | No | 3,384 | 97.1 | 100 | 2.9 | 2.01 | 0.1558 |  |
|  | Yes | 499 | 98.2 | 9 | 1.8 |  |  |  |
|  |  |  |  |  |  |  |  |  |
| Hypertension | No | 2,057 | 96.1 | 83 | 3.9 | 27.95 | <.0001 |  |
|  | Yes | 1,826 | 98.8 | 22 | 1.2 |  |  |  |
|  |  |  |  |  |  |  |  |  |
| Weight Status | Normal | 1,135 | 99.2 | 9 | 0.8 | 2.18 | 0.3365 |  |
|  | Overweight | 1,588 | 99.6 | 6 | 0.4 |  |  |  |
|  | Obese | 1,160 | 99.3 | 8 | 0.7 |  |  |  |
|  |  |  |  |  |  |  |  |  |
